# Diet/lifestyle and risk of diabetes and glycemic traits: a Mendelian randomization study

**DOI:** 10.1186/s12944-018-0666-z

**Published:** 2018-01-29

**Authors:** Renyu Ding, Tao Huang, Jiali Han

**Affiliations:** 1 Department of Otolaryngology, The First Hospital of China Medical University, Nanjing Bei Street 155, Shenyang, 110001 Liaoning Province People’s Republic of China; 2 Department of Epidemiology and Biostatistics, School of Public Health, Peking University, Beijing, 100191 China

## Abstract

**Background:**

Observational studies have demonstrated diet/lifestyle play roles in development of type 2 diabetes (T2DM); however, it remains unclear whether these relationships are causal.

**Methods:**

A two-sample MR approach was used to examine the causal effect of diet/lifestyle upon risk of T2DM and glycemic traits.

**Results:**

The protein intake-increasing allele C of *FTO* was significant associated with higher risk of T2DM (Beta ± SE = 0.104 ± 0.014, *P* = 4.40 × 10^− 11^), higher level of HOMA-IR (Beta ± SE = 0.016 ± 0.004, *P* = 9.55 × 10^− 5^), HOMA-B (Beta ± SE = 0.008 ± 0.003, *P* = 0.020). Using MR analyses, increased protein intake was causally associated with an increased risk of T2DM (Beta ± SE = 0.806 ± 0.260, *P* = 0.002). In addition, smoking cessation was causally associated with increased levels of glycemic traits such as HOMA-IR (Beta ± SE = 0.165 ± 0.072, *P* = 0.021), fasting insulin (Beta ± SE = 0.132 ± 0.066, *P* = 0.047) and fasting glucose (Beta ± SE = 0.132 ± 0.064, *P* = 0.039).

**Conclusions:**

These results provide evidence supporting a causal role for higher protein intake and smoking cession in T2DM. Our study provides further rationale for individuals at risk for diabetes to keep healthy lifestyle.

## Background

Diabetes has become a worldwide epidemic, with an estimated more than 387 million people with diabetes in 2014. However, the etiology of diabetes is poorly understood. Identifying potentially causal risk factors could help guide prevention efforts.

Compelling evidence showed that excessive caloric intake is a major driving force behind escalating obesity and type 2 diabetes (T2DM) epidemics, Importantly, diet quality of fats, carbohydrates and protein, [1] and unhealthy lifestyles have been also implicated in the rapid rise of T2DM [1]. For example, cigarette smoking is a well-established risk factor for T2DM, and smoking cessation leads to higher short-term risk [2]. In addition, both short and long sleep duration are associated with a significantly increased risk of T2DM [3]. However, it remains uncertain whether these relationshipsare causal since it is difficult to fully protect observational studies from bias due to reverse causation or confounding.

In the absence of high-quality RCT data, the principles of Mendelian randomization (MR) can be applied to strengthen or refute the causality of diet/lifestyle in T2DM etiology [4]. This approachis based on the principle that genetic variants are randomly allocated at meiosis, and consequently are independent of many factors that bias observational studies, such as confounding and reverse causation [4]. Therefore, MR is conceptually similar to an RCT.

Recent advances in GWAS have substantially improved our understanding of genetic roles in diet/lifestyle such as macronutrients, [5, 6] cigarette smoking, [7] smoking initiation, [7] smoking cessation, [7] sleep, [8] allowing us to use MR to estimate their causal effects. Therefore, we undertook an MR analysis using summary statistics from DIAGRAM for T2DM and GAGIC consortium for glycemic traits, respectively to estimate the causal effects of diet/lifestyle factors on risk of T2DM and glycemic traits.

## Methods

### SNP selection and data sources

The design of our study had three key components: 1) the identification of genetic variants to serve as instrumental variable (IV) for diet/lifestyle; 2) the acquisition of summary datafor the genetic variants from the DIAGRAM and MAGIC consortium; and 3) the estimate of causal effect of diet/lifestyleon T2DM and glycemic traits.

We searched the GWAS catalog to identify single nucleotide polymorphisms (SNPs) associated with diet/lifestyle [9]. We included all SNPs identified from original study reports for diet/lifestyle such as macronutrients, [5, 6] cigarette smoking, [7] smoking initiation, [7] smoking cessation, [7] sleep [8] in the GWAS catalog as potential IV. Effect estimates of these diet/lifestyle-associated SNPs on the risk of T2DM [10] and glycemic traits such as HOMA-IR, [11] HOMA-B, [11] fasting insulin, [11] fasting glucose, [11] Hba1c, [12] 2hGLU [13] were assessed using the summary statistics from the DIAGRAM consortium and MAGIC consortium, respectively. Summary statistics from these consortia can be downloaded at the following links: DIAGRAM consortium, http://diagram-consortium.org/; MAGIC consortium, https://www.magicinvestigators.org/.Cohorts participating in the DIAGRAM consortium and MAGIC consortium received ethics approval from local institutional review boards and informed consent from all participants.

We harmonized the data from the summary statistics of consortium [14]. First, we identified variants that do not share the same allele pair between datasets, and then correct this if possible. Second, we identified variants with unmatched effect and other alleles and then ‘flip’ their effect estimates and effect allele frequencies in only one of the datasets.

### MR estimates and statistical analyses

**Fig. 1:**
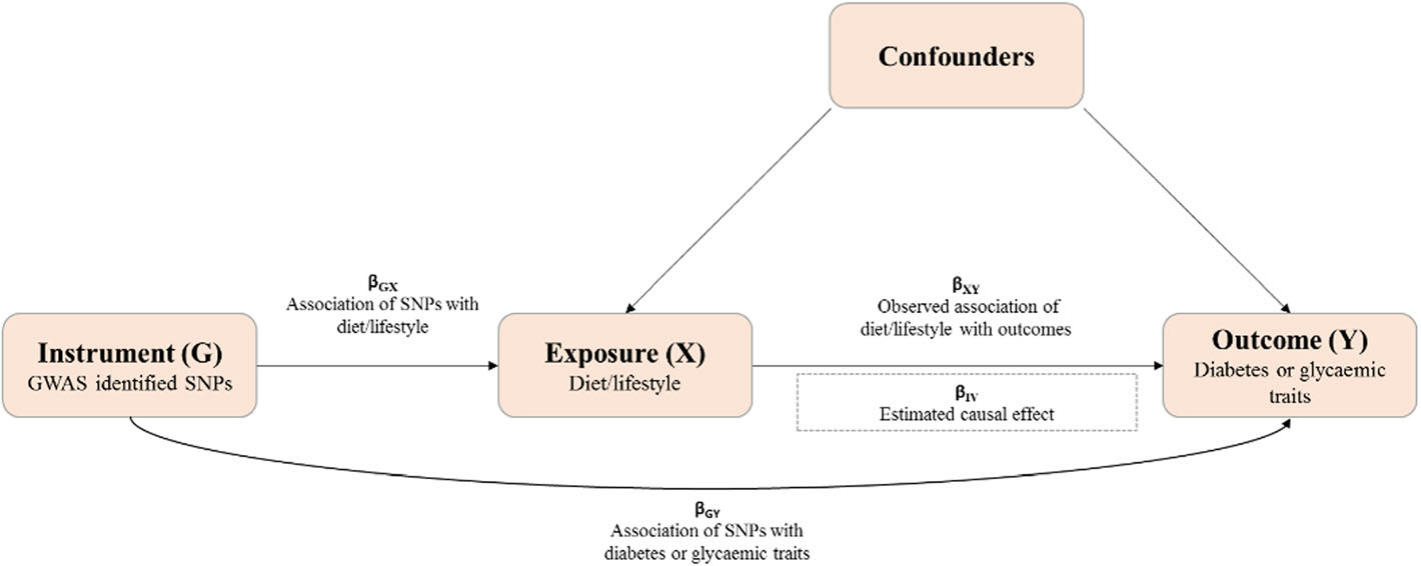
Schematic representation of an MR analysis. This diagram shows that SNPs associated with diet/lifestyle were selected from the GWAS studies. Corresponding effect estimates for these SNPs upon diabetes and glycemic traits were obtained from the DIAGRAM and MAGIC consortium. Because of the randomization of alleles at meiosis, SNPs are not associated with confounding variables that may bias estimates obtained from observational studies

Inference of causality in the estimated etiological associations between diet/lifestyle and risk of T2DM and glycemic traits is based on three MR assumptions [15]. The selected genetic variants are valid instrumental variables if these three assumptions are satisfied (Fig. 1). Three assumptions are a) the genetic variant (instrumental variable) is associated with exposure (diet/lifestyle); b) the genetic variant is not associated with confounders; and c) the genetic variant is associated with outcomes (T2DM and glycemic traits) only through their effect on exposure, not through other pathways.

Here, we employed the previously described methods to examine the causal association of diet/lifestyle with T2DM and glycemic traits [16]. We obtained the effect estimates of the selected SNPs on diet/lifestyle, and corresponding effect estimates for the selected SNPs on T2DM and glycemic traits were extracted from the DIAGRAM and MAGIC consortium. A two-sample MR approach were applied to examine causal effect. This two-sample approach has equivalent statistical power to one-sample approaches [17] and is favorable in this setting since large GWAS consortium and are thus better powered than an MR study in a single cohort with a smaller sample size. This approach weighted the effect estimate of each SNP on T2DM and glycemic traits by its effect on diet/lifestyle. These estimates were then pooled using a fixed meta-analytic model to produce a summary measure of the effect of diet/lifestyle on T2DM and glycemic traits [18].

*P* values were two-sided and evidence of association was declared at *P* < 0.05. Where indicated, Bonferroni corrections were used to make allowance for multiple testing, although this is likely to be overly conservative given the non-independence of many of the outcomes tested. All analyses were performed in R version 3.1.2 and Stata release 13.1 (StataCorp, College Station, TX).

## Results

### SNP selection

Overall, we identified 2, 2, 1, 8, 3, 1, 1that achieved genome-wide significance for carbohydrate intake, protein intake, fat intake, smoking, smoking initiation, smoking cessation, respectively. The SNPs for carbohydrate intake, protein intake, smoking, smoking initiation, and sleep duration are LD-independent SNPs. Therefore, in total we used 15 SNPs for our MR analysis, as shown in Table 1.

**Table 1:** Characteristics of single nucleotide polymorphisms used as instrumental variables

Lifestyle factors	Lifestyle-Associated SNP	Nearest Gene(s)	Chr	effect allele	Effect allele frequency	lifestyle		
						Beta	SE	P
Carbohydrate [5]	rs10163409	FTO	16	A	0.69	0.19	0.05	2.2 × 10^−4^
Carbohydrate [5]	rs197273	TANK	2	A	0.48	0.23	0.04	9.6 × 10^− 8^
Carbohydrate [6]	rs838145	IZUMO1	19	G	0.46	0.25	0.04	1.68 × 10^− 8^
Protein [6]	rs1421085	FTO	16	C	0.42	0.09	0.023	4.80 × 10^− 7^
Protein [5]	rs838133	FGF21	19	G	0.55	0.11	0.02	7.9 × 10^− 9^
Fat [6]	rs838145	IZUMO1	19	A	0.54	0.21	0.04	1.57 × 10^− 9^
Smoking (number) [7]	rs1051730	CHRNA3	15	A	0.35	1.021	0.056	2.75 × 10^− 73^
Smoking (number) [7]	rs1329650	LOC100188947	10	G	0.72	0.367	0.059	5.67 × 10^− 10^
Smoking (number) [7]	rs3733829	CYP2A6	19	G	0.36	0.333	0.058	1.04 × 10^− 8^
Smoking initiation [7]	rs6265	BDNF	11	C	0.79	0.061	0.011	1.84 × 10^− 8^
Smoking cessation [7]	rs3025343	DBH	9	G	0.84	0.121	0.022	3.56 × 10^− 8^
Sleep [8]	rs1191685	PAX8	2	C	0.37	2.87	0.47	1.06 × 10^− 9^
Sleep [8]	rs2394403	CBWD	6	C	0.8	3.07	0.56	4.39 × 10^−8^
Sleep [8]	rs4248149	CBWD	6	T	0.8	3.08	0.56	3.95 × 10^− 8^
Sleep [8]	rs4587207	CBWD	6	A	0.8	3.14	0.56	2.02 × 10^− 8^

Smoking initiation (ever versus never smokers); Smoking cessation (former versus current smokers)

### Association of the selected SNPs with diet/lifestyle

Table 1 displays the SNPs that identified in GWAS for diet/lifestyle and describes their associations with carbohydrate intake, [6] protein intake, [5, 6] fat intake, [6] smoking (number), [7] smoking initiation, [7] smoking cessation, [7] and sleep [8]. Each of these SNPs explained an important proportion of the population-level variance in diet/lifestyle.

### Association of the selected SNPs with T2DM and glycemic traits

We found that protein intake SNP rs1421085 at *FTO* was significantly associated risk of T2DM and glycemic traits (Table 2). The protein intake-increasing allele C of *FTO* was significant associated with higher risk of T2DM (Beta ± SE = 0.104 ± 0.014, *P* = 4.40 × 10^− 11^), higher level of HOMA-IR (Beta ± SE = 0.016 ± 0.004, *P* = 9.55 × 10^− 5^), HOMA-B (Beta ± SE = 0.008 ± 0.003, *P* = 0.020), Hba1c (Beta ± SE = 0.008 ± 0.004, *P* = 0.028), whereas lower level of fasting insulin (Beta ± SE = − 0.015 ± 0.004, *P* = 7.48 × 10^− 5^). In addition, the G allele of SNP rs3025343 at DBH forsmoking cessation was significantly associated with higher level of HOMA-IR (Beta ± SE = 0.020 ± 0.008, *P* = 0.010), fasting insulin (Beta ± SE = 0.016 ± 0.008, *P* = 0.032), and fasting glucose (Beta ± SE = 0.016 ± 0.007, *P* = 0.029).

**Table 2:** Lifestyle associated SNPs and glycemic traits

Lifestyle	Lifestyle-Associated SNP	T2D [10]		HOMA-IR [11]		HOMA-B [11]		INS [11]		GLU [11]		Hba1c [12]		2hGLU [13]	
		Beta ± SE	p	Beta ± SE	p	Beta ± SE	p	Beta ± SE	p	Beta ± SE	p	Beta ± SE	p	Beta ± SE	p
Carbohydrate	rs10163409	0.020 ± 0.018	0.370	0.006 ± 0.005	0.201	0.002 ± 0.004	0.580	0.004 ± 0.005	0.327	0.004 ± 0.004	0.309	− 0.008 ± 0.004	0.041	0.015 ± 0.022	0.495
Carbohydrate	rs197273	0.000 ± 0.015	0.970	−0.002 ± 0.004	0.606	− 0.005 ± 0.003	0.159	− 0.002 ± 0.004	0.667	0.003 ± 0.004	0.500	0.002 ± 0.004	0.609	0.021 ± 0.019	0.280
Carbohydrate	rs838145	0.020 ± 0.017	0.230	−0.002 ± 0.004	0.716	0.003 ± 0.003	0.405	−0.001 ± 0.004	0.879	0.001 ± 0.004	0.720	0.005 ± 0.004	0.165	−0.013 ± 0.020	0.498
Protein	**rs1421085**	0.104 ± 0.014	**4.40 × 10–11**	0.016 ± 0.004	**9.55 × 10–5**	0.008 ± 0.003	**0.020**	−0.015 ± 0.004	**7.48 × 10–5**	0.007 ± 0.004	0.053	0.008 ± 0.004	**0.028**	−0.001 ± 0.019	0.981
Protein	**rs838133**	0.010 ± 0.018	0.720	0.002 ± 0.005	0.626	0.002 ± 0.004	0.546	0.001 ± 0.004	0.904	0.001 ± 0.004	0.805	−0.006 ± 0.004	0.153	0.013 ± 0.021	0.534
Fat	rs838145	−0.020 ± 0.017	0.230	0.002 ± 0.004	0.716	0.003 ± 0.003	0.405	0.001 ± 0.004	0.879	−0.001 ± 0.004	0.720	−0.005 ± 0.004	0.165	0.013 ± 0.020	0.498
Sleep	rs1191685	−0.010 ± 0.015	0.370	0.002 ± 0.005	0.655	0.004 ± 0.004	0.270	0.002 ± 0.005	0.701	−0.005 ± 0.004	0.225	−0.006 ± 0.004	0.119	0.028 ± 0.021	0.184
Sleep	rs2394403	0.030 ± 0.015	0.094	0.002 ± 0.005	0.685	0.003 ± 0.005	0.464	0.001 ± 0.005	0.840	−0.002 ± 0.005	0.676	−0.003 ± 0.005	0.482	0.002 ± 0.026	0.943
Sleep	rs4248149	0.030 ± 0.015	0.076	0.002 ± 0.006	0.783	0.002 ± 0.005	0.655	0.000 ± 0.005	0.934	−0.001 ± 0.005	0.821	−0.002 ± 0.005	0.747	−0.005 ± 0.027	0.849
Sleep	rs4587207	0.010 ± 0.015	0.670	0.001 ± 0.005	0.798	0.003 ± 0.005	0.456	0.000 ± 0.005	0.937	−0.003 ± 0.005	0.552	−0.003 ± 0.005	0.506	−0.002 ± 0.026	0.940
smoking (number)	rs1051730	−0.030 ± 0.017	0.035	0.003 ± 0.004	0.459	−0.002 ± 0.004	0.657	0.002 ± 0.004	0.663	0.003 ± 0.004	0.430	0.004 ± 0.004	0.281	0.001 ± 0.020	0.951
smoking (number)	rs1329650	0.010 ± 0.013	0.290	−0.008 ± 0.004	0.055	−0.004 ± 0.004	0.300	−0.006 ± 0.004	0.169	−0.005 ± 0.004	0.192	−0.005 ± 0.004	0.226	0.007 ± 0.020	0.742
smoking (number)	rs3733829	0.010 ± 0.015	0.410	0.002 ± 0.004	0.713	−0.002 ± 0.004	0.595	−0.001 ± 0.004	0.752	0.003 ± 0.004	0.418	0.003 ± 0.004	0.362	−0.016 ± 0.020	0.444
Smoking initiation	rs6265	0.010 ± 0.015	0.500	0.005 ± 0.005	0.346	0.003 ± 0.004	0.494	0.004 ± 0.005	0.392	0.001 ± 0.005	0.793	0.009 ± 0.004	0.042	−0.033 ± 0.024	0.166
Smoking cessation	**rs3025343**	0.068 ± 0.038	0.099	0.020 ± 0.008	**0.010**	0.008 ± 0.007	0.233	0.016 ± 0.008	**0.032**	0.016 ± 0.007	**0.029**	0.011 ± 0.007	0.093	0.038 ± 0.039	0.330

### MR estimates

Using MR analyses, we found that increased percentages of total energy consumption from protein was causally associated with an increased risk of T2DM (Beta ± SE = 0.806 ± 0.260, *P* = 0.002) (Table 3). TheI^2^estimate for heterogeneity was 0% (95% CI 0% -12%). However, increased percentages of total energy consumption from protein was not associated with glycemic traits (Table 3). In addition, our results also suggested that smoking cessation was causally associated with increased levels of glycemic traits such as HOMA-IR (Beta ± SE = 0.165 ± 0.072, *P* = 0.021), fasting insulin (Beta ± SE = 0.132 ± 0.066, *P* = 0.047) and fasting glucose (Beta ± SE = 0.132 ± 0.064, *P* = 0.039). However, we did no observe significant association between fat intake, carbohydrate intake, sleep duration, and smoking initiation and number of cigarettes and risk of T2DM and glycemic traits (Table 3).

**Table 3:** Results of MR analyses of lifestyle and glycemic traits

Lifestyle	MR Estimates													
	T2D		HOMA-IR		HOMA-B		INS		GLU		Hba1c		2hGLU	
	Beta ± SE	p	Beta ± SE	p	Beta ± SE	p	Beta ± SE	p	Beta ± SE	p	Beta ± SE	p	Beta ± SE	p
Carbohydrate	0.051 ± 0.045	0.262	0.000 ± 0.011	0.981	0.000 ± 0.009	0.990	0.001 ± 0.010	0.957	0.011 ± 0.010	0.282	0.003 ± 0.010	0.745	0.029 ± 0.052	0.582
Protein	**0.806 ± 0.260**	**0.002**	**0.067 ± 0.035**	**0.046**	0.045 ± 0.027	0.092	−0.047 ± 0.033	0.157	0.038 ± 0.029	0.203	0.049 ± 0.041	0.231	0.062 ± 0.141	0.662
Fat	−0.079 ± 0.070	0.261	0.007 ± 0.020	0.722	0.014 ± 0.016	0.400	0.003 ± 0.019	0.881	−0.007 ± 0.018	0.713	−0.020 ± 0.015	0.175	0.062 ± 0.095	0.519
Sleep	0.005 ± 0.003	0.058	0.001 ± 0.001	0.487	0.001 ± 0.001	0.128	0.000 ± 0.001	0.713	−0.001 ± 0.001	0.222	−0.001 ± 0.001	0.106	0.003 ± 0.004	0.534
Smoking cessation	0.559 ± 0.328	0.090	**0.165 ± 0.072**	**0.021**	0.064 ± 0.055	0.247	**0.132 ± 0.066**	**0.047**	**0.132 ± 0.064**	**0.039**	0.093 ± 0.058	0.107	0.314 ± 0.326	0.337
Smoking initiation	0.249 ± 0.380	0.515	0.079 ± 0.085	0.353	0.048 ± 0.071	0.503	0.069 ± 0.081	0.397	0.020 ± 0.077	0.799	0.141 ± 0.073	0.055	−0.541 ± 0.403	0.182
smoking (number)	−0.013 ± 0.014	0.357	0.001 ± 0.004	0.835	−0.003 ± 0.003	0.367	0.000 ± 0.004	0.914	0.002 ± 0.004	0.575	0.003 ± 0.003	0.379	−0.001 ± 0.018	0.944

We pooled β coefficients across SNPs using fixed-effect meta-analysis. After meta-analysis, we used the IV estimators toquantify the strength of the causal association of diet/lifestyle anddiseases. The IV estimator which is identical to that derived by the widely used two-stage least squares method, will be calculated as the β of the regression coefficients SNP-exposure and SNP-outcome

## Discussion

Using a MR study design, we found that genetically elevated protein intake and smoking cession are causally associated with an increased risk of T2DM and higher level of insulin resistance. These results provide evidence supporting a causal role for higher protein intake and smoking cession in diabetes, and suggest that individuals at risk for diabetes to keep healthy lifestyle.

Previous research efforts have focused on macronutrient intake in relation to type 2 diabetes risk, [19] but mainly on relative carbohydrate and fat content. Meanwhile, high-protein diets may contribute to disturbance of glucose metabolism. Previous studies addressing dietary protein and diabetes risk focused mainly on high-protein food groups, such as meat and soy. For example, red processed meat intake was related to increased diabetes risk [20–22]. In addition, another study found an increased diabetes risk with higher intake of animal protein and no association with vegetable protein intake, [23] whereas intake of legumes and soy decreased diabetes risk in Asians [24]. However, the Nurses Health Study II did not find such an association, [25] suggesting divergent effects of animal and vegetable protein. The contradictory findings may reflect reverse causation bias and confounding effects. Interestingly, the European Prospective Investigation into Cancer and Nutrition (EPIC)-NL study found that high total protein intake was associated with increased diabetes risk, [22] and suggested that this relation was not explained by specific protein sources such as meat [22]. However, the causality between protein intake and risk of diabetes is not documented. Furthermore, several trials showed beneficial effect of high protein intake on glycemic traits, [26, 27] whereas, high protein diet did not decrease hemoglobin A1c and fasting plasma glucose, and increase insulin sensitivity, [28] making the conclusion difficulty. In the present MR analysis, we found that protein intake was causally associated with increased risk of diabetes and insulin resistance. Our results were generated by using the large scale GWAS summary results, which suggest the robustness of our findings. Our findings for protein intake are generally consistent with those based on prospective observational studies, which tend to report increased risk of diabetes. In addition, reliable GWAS identified genetic variants for protein intake were used as instrumental variables to infer the causality, therefore, our findings were protected from bias such as confounding and reverse causation [4].

Cigarette smoking is an established predictor of incident T2DM. Therefore, smoking cessation should be associated with a decrease in the risk of T2DM. However, smoking cessation is associated with substantial weight gain, [29] which could increase diabetes risk. Several studies have found an increased diabetes risk after smoking cessation [2, 30–32]. However, residual confounding is possible even with meticulous adjustment for established diabetes risk factors in the observational studies. Interestingly, a systematic review and meta-analysis of data from randomised controlled trials of smoking cessation in adults with diabetes found that pooled results did not provide evidence of efficacy for smoking cessation interventions in people with diabetes [33]. Therefore, the causality between smoking cessation and risk of diabetes are still unknown. Using GWAS identified genetic variants for smoking cessation as instrumental variable, our MR analysis provided robust evidence to support that smoking cessation might cause increased risk of T2DM. These findings may carry important public health implications. Smokers at risk for diabetes who quit should receive advice about avoiding weight gain and about diabetes prevention and early detection.

However, our MR did not observe causal association of other lifestyle factors such as fat and carbohydrate intakes and number of smoking cigarettes, smoking initiation, sleep duration, and morning person with risk of T2DM. These findingsare generally contradictory to those based on previous prospective studies, which tend to report increased risk for diabetes in individuals with smoking, both shorter and longer sleep. The contradictory findings may reflect confounding effects, e.g. due to cases being slightly older than controls, or reverse causation bias in the retrospective studies, whereby lifestyle changes arise as a result of disease.

Given the random distribution of genotypes in the general population with respect to lifestyle and other environmental factors, the results of the MR analysis may offer some of the best evidence to assess the causal role of protein and smoking cession in T2DM etiology since the results are less likely to be biased by confounding or reverse causation than traditional observational epidemiological study designs. By employing the two-sample MR approach, we were able to increase statistical power by selecting summary statistics from the largest GWAS studies for T2DM (DIAGRAM, *n* = 149,821) and glycemic traits (MAGIC, *n* = 133,010).

Our study is subject to some limitations. First, our results assume that the samples used to define the instrumental variable for diet/lifestyle and the samples from consortium used to estimate the genetic association with disease/traits are from the same population. Second, our results mightbeconfoundedby pleiotropic pathways.^35^We cannot entirely rule out this possibility. Third, our study assumed a linear relationship between diet/lifestyle and T2DM and glycemic traits. In this study, we could not investigate nonlinear effects of diet/lifestyle. Fourth, we cannot exclude the possibility of sample overlap since both DIAGRAM and the MAGIC consortiums used samples from the lifestyle GWASstudy. Therefore, this might introduce bias into our results. Finally, we cannot rule out chance of violation of any of the three MR assumptions, which would potentially bias the magnitude of the estimated causal association [34].

## Conclusions

In summary, these results provide evidence supporting a causal role for higher protein intake and smoking cession in diabetes. This provides further rationale for individuals at risk for diabetes to keep healthy lifestyle. However, whether different sources of protein diet, or duration of smoking cession, is mediating these relationships warrants further investigations.
